# Nervous Diseases and Insanity

**Published:** 1896-05

**Authors:** James Wright Putnam, William C. Krauss

**Affiliations:** Clinical professor of diseases of the nervous system, Buffalo University Medical College; Professor of nervous diseases in Niagara University Medical College


### NERVOUS DISEASES AND INSANITY.

CONDUCTED BY JAMES WRIGHT PUTNAM, M.D.,
Clinical professor of diseases of the nervous system, Buffalo University Medical College, and WILLIAM C. KRAUSS, M.D.,
Professor of nervous diseases in Niagara University Medical College.

### GONORRHEAL PSYCHOSES.

PROF. S. VENTURI (*Hospitals-Tidende*, No. 42, 1894, quoted in *Lancet-Clinic*,) calls attention to the relation between gonorrhea and certain abnormal mental conditions. Among twenty-two patients with hebephrenia, in his institution, there were twelve with a gonorrheal discharge, and, in contrast, with true hebephrenia the disease was cured in four to eight months, a shorter or longer time after the cure of the gonorrhea. The disease would appear one to six months after infection and be characterised by stupor, with anxious hallucinations, intermittent delirium and attempts at suicide, insomnia, general hyperesthesia and augmented cutaneous reflexes. No fever. In occasional cases there were cataleptic and catatonic states, choreiform movements and maniacal excitement of longer or shorter duration. He assumes an infection of the meninges by the gonococcus. Balsamics are indicated in its treatment.

---

### ACUTE NONSUPPURATIVE ENCEPHALITIS.

OPPENHEIM (*Centralbl. f. klin. Med.*; quoted in *Medical Review*,) says the separation of the nonsuppurative from the suppurative disease is most important. The etiology of the hemorrhagic form of the disease is not always the same. By some it has been attributed to alcohol, by others to influenza and there are a number of cases of no known etiology. It begins with severe symptoms, but usually runs a favorable course. He gives short details of five cases observed by him, all of which recovered. Three cases were acute, occurring respectively in girls aged 16 and 10 and in a young woman. In two subacute cases, in a girl aged 12 and a man aged

21, the lesion lay in the floor of the fourth ventricle (Wernicke's type), and a complete ophthalmoplegia developed itself. In none of these cases was there any evidence of syphilis. In the literature of this disease the prognosis has not always been looked upon as very favorable, but in recent writings recoveries have been noted. His experience has been most favorable. Polioencephalitis must be distinguished from disseminated sclerosis and which, according to him, may end in complete recovery. Acute course, rapid development, high temperature and the like, are unfavorable signs, whereas low temperature and a protracted course make the outlook favorable.—*British Medical Journal.*

---

### CLAIMED TEST FOR INSANITY.

Dr. Burton Ward declares (*Medical Age*) there is one infallible symptom indicating whether one is sane or not. Let a person speak ever so rationally and act ever so sedately, if his or her thumbs remain inactive there is no doubt of insanity. Lunatics seldom make use of their thumbs when writing, drawing or saluting.

---

### THE SPINAL CORD.

Irritation of the spinal cord (*Medical Age*) increases secretion. Congestion of the lumbar plexus or of the cord will cause diabetes; a little lower down, diarrhea. Congestion of the cervical plexus causes vomiting and palpitation.

---

### INSANITY AND MURDER.

The Supreme Court of the United States has (*Medical Record*) just handed down a decision which is of much importance in cases involving a question of the insanity of murderers. The court has reversed the decision of the inferior court and lays down the principle that the burden of proof of guilt rests in all criminal cases upon the prosecution ; in other words, the prosecuting attorney, in cases where the claim of insanity is put up, must prove that the accused is not insane.

---

### IDIOTS' SKULLS.

Sir George Humphrey reports (*Medical Age*), after examining nineteen specimens of idiots' skulls, that he is unable to find anything suggesting that deficiency in development is the leading feature in the deformity, or that the smallness of the bony cerebral envelope exerts a depressing or dwarfing influence upon the brain—nothing, in fact, to encourage the practice recently advocated of removing part of the calvarium with an idea of affording more space and freedom for the growth of the brain.

---

### ALTERATIONS OF THE SPINAL CORD IN PERNICIOUS ANEMIA.

In an inaugural dissertation (Stockholm, 1895, Eklund, *Univ. Med. Journal,*) Charles Petren states that in pernicious anemia small hemorrhages and consecutive sclerosis are frequently met with in the spinal marrow. These hemorrhages have no significance from a clinical point of view. The vessels often show thickening and commencing hyaline degeneration, not, however, as a rule, combined with degeneration of the nervous elements. From a study of the literature it appears that comparatively few cases of pernicious anemia present a real disease of the spinal cord. The symptoms of anemia remain unchanged in cases in which it does occur, and it is difficult to explain why the cord should be affected in some cases and not in others. The disease of the cord manifests itself with somewhat varying symptoms, certain of which, however, are exhibited in all cases. From an anatomical point of view, the alterations have considerable variations, but this is accounted for to a great extent by the fact that the process has been observed at a different stage in the various cases; from a closer analysis of the cases it appears that the degeneration progresses in a fairly regular manner. It is presumable that these cases of disease of the spinal cord form a special group, even from a neurological point of view. It may be admitted that some toxic condition is the common, immediate cause of the disease of the spinal marrow as well as of the anemia. The alterations of the spinal cord are here wholly different from those found in tuberculosis and diabetes, where the changes can be easily distinguished by slightly-marked and chronic degeneration, such as is often found in Addison's disease.

---

### EPILEPTIFORM INEBRIETY.

Dr. T. D. Crothers, (*Lancet-Clinic,*) in describing the paroxysmal outbreaks of the inebriate and his innocence or guilt in certain criminal cases, gives the following conclusions in determining the facts of the case :

1. The periodicity of the drink attacks.

2. The mental conditions which preceded or followed them.

3. The character and conduct of the case in the free interval for purposes of comparison.

4. The act in question and the time and condition of the man when it was committed.

5. The facts of heredity and his probable degree of mental vigor and health, together with his present state of mind and body.

A study of these facts will most naturally bring out a clear conception of the mental condition of the man and his degree of sanity and soundness, with consciousness of the act and power of control at the time.

---

### THE ALLEGED REFLEX CAUSES OF NERVOUS DISEASE.

Philip Coombs Knapp (*Am. Med.-Sur. Bull.*) arrives at the following conclusions:

1. The essential feature in the production of many neuroses is the neuropathic state, degeneracy, of the subject.

2. In hysterical subjects, suggestion plays an important part, both in the development and cure of the symptoms.

3. Disease of any organ may give rise to referred pain in some definite area, but not to other nervous disturbances, except as a secondary result of local diseases of the organ. This local disease manifests itself by the ordinary local symptoms and the nervous phenomena are due to exhaustion, anemia, intoxication, and the like.

4. In a few rare cases, injury of a sensory nerve may give rise to epileptiform seizures.

5. Surgical operations for the relief of nervous symptoms should never be performed unless there are clear indications, apart from such symptoms, for an operation.

